# Prevalence of Hepatitis B Virus Infection among Pregnant Women Attending Antenatal Clinics in Vientiane, Laos, 2008–2014

**DOI:** 10.1155/2017/1284273

**Published:** 2017-03-29

**Authors:** Marc Choisy, Sengdeuane Keomalaphet, Kinnaly Xaydalasouk, Fabrice Quet, Vatthanaphone Latthaphasavang, Yves Buisson

**Affiliations:** ^1^MIVEGEC, Université de Montpellier, CNRS 5290, IRD 224, Montpellier, France; ^2^Oxford University Clinical Research Unit, Hanoi, Vietnam; ^3^Institut de la Francophonie pour la Médecine Tropicale (IFMT), Vientiane, Laos; ^4^Department of Infectious Diseases, Mahosot Hospital, Vientiane, Laos

## Abstract

The Lao People's Democratic Republic (PDR) is still considered a highly endemic country for hepatitis B, mainly due to perinatal transmission of hepatitis B virus (HBV), despite efforts made since 2004 for universal immunization of newborns. The prevalence of HBV surface antigen (HBsAg) carriage in pregnant women is a relevant marker for the risk of mother-to-child HBV transmission. This study aimed to assess the changes in prevalence of HBV infection among pregnant women attending the Mahosot Prenatal Clinic (Vientiane Capital).* Methods*. A retrospective study was performed in the Mahosot Hospital Laboratory to collect and analyze all the results of HBsAg testing in pregnant women from 2008 to 2014.* Results*. Of a total of 13,238 tested women of mean age of 26 years, 720 women (5,44% [95 CI: 5.1–5.8%]) were found HBsAg positive, the annual prevalence ranging from 4.6% to 6.2%. A slight but steady and significant decrease in prevalence over the 7 years of the study could be documented.* Conclusion*. Although below the 8% hyperendemic threshold, the HBsAg prevalence observed in pregnant women in Vientiane reflects a high risk of HBV perinatal transmission and call for a widespread infant immunization with an HBV vaccine birth dose.

## 1. Background

The Lao People's Democratic Republic (PDR) is assumed to be highly endemic for hepatitis B. The prevalence rate of chronic infection with hepatitis B virus (HBV) was estimated at 8.7% in 13,897 blood donors in 2003–2005 [1]. As in other countries in Southeast Asia, the mother-to-child transmission is considered the main route of infection: a seroepidemiological study conducted in 2011 among 398 pregnant women in two major cities, Luang Prabang and Vientiane, revealed a seroprevalence of antibodies anti-HBc and HBV surface antigen (HBsAg) equal to 49.5% and 8.2%, respectively [2]. The former marker reflects past exposure, whereas the latter reflects current infections.

The occurrence of HBV infection early in life increases the risk of progression to chronic liver disease, the development of cirrhosis, and hepatocellular carcinoma. It is now well accepted that universal immunization of newborns is the most effective way to eliminate hepatitis B [3]. The first strategy was to vaccinate all children born to mothers infected with HBV. It involves a systematic screening for HBV markers of infection (HBsAg) and replication (HBeAg) in pregnant women, followed, if positive, by active-passive immunization of the newborn within the first 24 hours of life, combining hepatitis B vaccine and hepatitis B immunoglobulin (HBIG) injected in two different sites. This strategy may reduce by 75–90% the mother-to-child HBV transmission [4], but it is difficult to generalize in highly endemic areas because of its high cost. For low-income countries, universal vaccination strategy for children without prenatal screening is the only way to control HBV infection and prevent its long-term sequelae [5]. In line with WHO recommendations, vaccination against hepatitis B was introduced in the expanded program on immunization (EPI) of the Lao PDR in 2001. In 2004, the administration of a birth dose was introduced to lower, by 2012, the prevalence of HBsAg under the threshold of 2% in children under 5 years [6]. However in 2011, only 34% of infants had received a birth dose of hepatitis B vaccine for the reason that most Lao women gave birth at home without medical assistance [7]. The offer of free vaccination of newborns during the postpartum in the nearest health center or in the district hospital is rarely exploited. The situation appears more favourable in urban areas where women have better access to health facilities. In 2001, a serological screening of HBsAg among the pregnant women has been initiated in the prenatal clinic of the Mahosot Hospital, the main university hospital in Vientiane capital, as a relevant indicator for the risk of maternal-foetal transmission of HBV. The test is performed for all women attending antenatal clinics for 45,000 LAK (about 5.5 US dollars). This study aimed to analyze the HBsAg testing results collected for seven consecutive years to estimate the residual risk of perinatal HBV infection in Vientiane.

## 2. Methods

### 2.1. Study Population

A retrospective study was conducted at the laboratory of the Mahosot Hospital where the HBsAg screening tests are performed. It covered all archived results from pregnant women attending the antenatal clinic, available from 2008: 1684 results in 2008, 1830 in 2009, 2023 in 2010, 2209 in 2011, 1894 in 2012, 1154 in 2013, and 2444 in 2014. The only usable data were the age of women, the date of blood sampling, and the result of HBsAg testing. Women who have had multiple deliveries during the study period underwent HBsAg screening every time. Considering that every pregnancy is a risk of mother-to-child transmission, duplicates have not been removed.

### 2.2. Detection of HBsAg

The venous blood samples were taken at the antenatal clinic and sent the same day to the hospital laboratory for testing. During the early years, HBsAg was detected by an immunochromatographic 1-step test, the HEXAGON HBsAg (Human GmbH, Wiesbaden, Germany). Since December 2012, the detection of HBsAg was made with the ELISA kit HBsAg (Human GmbH, Wiesbaden, Germany) following the instructions provided by the manufacturer. Doubtful or indeterminate results were excluded from the analysis.

### 2.3. Data Analysis

The temporal trend of the mean age of pregnant women attending Mahosot Hospital was modeled by a linear regression model. The temporal trend of the prevalence and the age effect on the prevalence were modeled by a logistic regression model using time and age as explanatory variables and the positivity to HBsAg as the response variable. Naturally, we expect the HBsAg positive rate to decay with age. Collinearity between age and time variables may lead to confounding effects affecting the significance tests on these two variables. In order to correct for such confounding effects, we employed sequential likelihood ratio tests as suggested by Faraway [8]. Time × age interaction and polynomial terms (up to degree 3) for explanatory variables were considered and tested.

The overall level of seroprevalence in this study was compared with levels of seroprevalence observed in two other surveys recently conducted in Laos by Fisher's exact tests.

All analyses were done with the R software, version 3.3.0 [9].

## 3. Results

**Figure 1:**
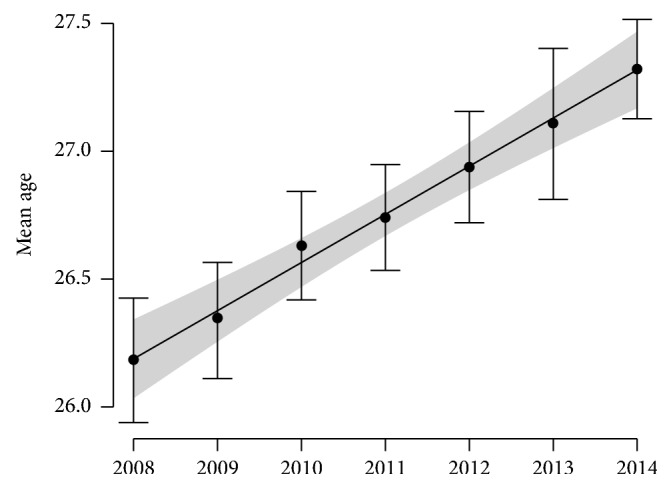
Increase of the mean age of pregnant women attending Mahosot Hospital from 2008 to 2014. Dots and vertical bars show estimates and their 95% confidence intervals from data. Black curve and grey area show the prediction of the linear regression model and its 95% confidence interval.

A total of 13,238 pregnant women were tested for HBsAg during the seven years from 2008 to 2014 (mean: 1891 ± 380 women per year). Their mean age was 26 years (SD 4.96, range 13–48 years) and steadily increased from 26.18 (standard error: 0.12) years in 2008 to 27.32 (SE: 0.10) years in 2014 at a constant rate of 2.261 (95 CI: 1.755–2.767) month per year (*F* = 76.74, df = 1 and 13,237, *p* < 2.2*e* − 16); see Figure 1.

**Figure 2:**
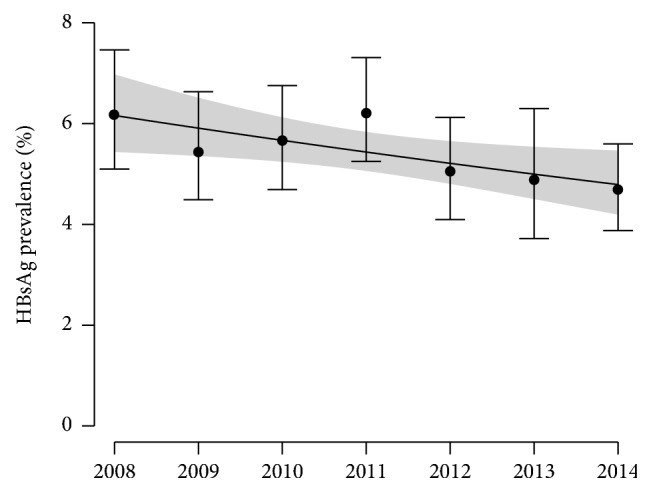
HBsAg prevalence as a function of time. Dots and vertical bars show estimates and their 95% confidence intervals from data. Black curve and grey area show the prediction of the logistic regression model and its 95% confidence interval.

HBsAg carriage was detected in 720 pregnant women, corresponding to an overall rate of 5.44% (95 CI: 5.1–5.8). The logistic regression model showed a significant general decrease of the prevalence from 2008 to 2014 (Chi-square = 5.1992; df = 1; *p* = 0.0226; Table 1 and Figure 2) with a relative decrease of prevalence of 4.438%/year (95 CI: 0.673–8.218%). Age did not have any significant effect on the prevalence (see Table 1), neither did the age × time interaction (Chi2 = 2.0197; df = 1; *p* = 0.1553). Furthermore, polynomial terms (up to degree 3) did not increase the fit significantly (Chi2 = 0.5387, df = 1, *p* = 0.4630 for degree 2; Chi2 = 0.0766, df = 1, *p* = 0.7820 for degree 3). In consequence, Table 1 shows the final selected model without interaction and without polynomial terms.

## 4. Discussion

The main objective of this study was to evaluate the potential risk of mother-to-child HBV transmission from routine screening results conducted in pregnant women at the antenatal clinic in Vientiane. The secondary objective was to determine whether this risk has remained stable or if it has changed over the past seven years.

The first observation is that the overall prevalence rate of HBsAg carriage, equal to 5.44% among these pregnant Lao women, is lower than the 8% threshold that defines the high endemicity level [10]. Interestingly, large differences are observed when comparing it to the results of two recent surveys conducted in Laos: it is significantly lower than the 8.2% rate reported among 388 pregnant women tested in 2011 in Luang Prabang and Vientiane [2] but significantly higher than the 2.9% rate established in a 2012 nationwide survey conducted on 965 mothers [11]. How can such discrepancies be explained? By analyzing these two surveys, some differences in sociodemographic characteristics of the study populations can be noted. Our study population is similar to that recruited in 2011 [2], but it greatly differs from the nationwide prevalence survey conducted on a probability sampling of mothers of whom 71% were farmers [11]. These data support the hypothesis of an inhomogeneous distribution of HBV infection between the urban (33%) and rural (67%) components of the population of Lao PDR [12]. In contrast to observations that show HBsAg carriage rates are significantly higher in rural than in urban areas, such as in Mongolia [13] or Vietnam [14], Laos' situation would be comparable to that of northern Gabon where HBsAg prevalence (12.9% in urban areas versus 7.6% in rural areas) seems to be linked to high population density in the main city and significant population movements related to trade with neighboring countries [15]. Whether the urban populations are more heavily exposed to sources of HBV infection or more extensively screened for HBsAg carriage would deserve to be clarified. Further studies are needed to specify the prevalence of HBV infection in Laos according to region, ethnicity, and rural or urban lifestyles.

Another important result of this retrospective study is to highlight a decreasing trend in the HBsAg prevalence rates among pregnant women consulting in Vientiane capital that is modest but significant. This trend cannot be credited to the routine HBV immunization of infants, the first beneficiaries of which have not yet reached the age of 15 years. Indeed, the HBsAg positivity rate does not appear lower in younger age groups, unlike a similar retrospective study in Thailand, where vaccination of newborns was introduced in 1992, showing a significant decrease of HBsAg prevalence in pregnant women under the age of 20 years [16]. Furthermore, despite a concurrent increasing trend in the mean age of pregnant women attending Mahosot Hospital, the logistic regression revealed that this increasing trend in mean age had no effect on the decreasing trend in prevalence.

In Hong Kong, the slight decrease of the HBsAg prevalence in pregnant women observed between 1983 and 1995 was explained by several factors besides vaccination: the reduction of the risk of nosocomial transmission by using disposable syringes, the systematic screening of blood donors, and the behavioral changes induced by the AIDS prevention program [17]. In Laos, blood safety has been improved with routine screening of HBsAg in donors performed by the National Blood Transfusion Center (Lao Red Cross, Vientiane) and Control Programs against HIV infection/AIDS have been implemented by the National committee for the control of AIDS (NCCA) established in 1988, but these measures have no direct impact on perinatal transmission. Despite the remarkable progress made by the Lao PDR to achieve the WHO objectives of hepatitis B control, it is likely that the decrease of HBsAg prevalence will be much faster when girls vaccinated at birth will be of childbearing age, that is, from 2025 onwards. However, several solutions should be considered in order to enhance the current incomplete immunization coverage of children, such as strengthening mobile vaccination teams in remote areas or providing an additional dose of HBV vaccine at 10 years of age [2].

Several limitations should be considered. First, despite its large size, the study population is not a representative sample of the whole Lao population. Indeed, these are women living in urban areas in the capital of Laos, having the financial ability to pay for antenatal care and HBsAg screening. However, the homogeneity of this recruitment for seven consecutive years allows using this population as a relevant indicator of the evolutionary trend of the HBV endemic status. Several studies have shown that low socioeconomic status was a risk factor for HBV infection [18]. So we can assume that pregnant women who do not attend the antenatal clinic and cannot undergo the HBV screening for lack of economic means have a higher HBsAg prevalence.

Another limitation is that laboratory tests have been limited to the detection of HBsAg, without checking the markers of viral replication, as HBe antigen or HBV DNA, for economic reasons. The presence of HBeAg in HBsAg positive pregnant women increases the risk of perinatal transmission of HBV. In Southeast Asia, over 30% of the HBsAg positive women are presumed to remain HBeAg carriers between 20 and 39 years, therefore at high risk of transmission [19].

The HBsAg detection method can also be questioned because it used a rapid chromatographic immunoassay for the first 5 years (2008–2012) and then ELISA (2013-2014). The performance of the rapid test HEXAGON HBsAg has been evaluated in Madagascar at 95.6% for sensitivity and 96.3% for specificity [20]. All HBsAg rapid tests have in common a low sensitivity that makes them unsuitable for HBsAg detection at low concentrations. The analytical sensitivity of the ELISA test used, estimated <0.13 International Units/milliliter, is 5 to 10 times higher than that of rapid tests [21]. Thus, the substitution by ELISA at the end of 2012 could have been followed by an increasing prevalence of HBsAg, but this was not observed. On the contrary, despite the use of a more sensitive test in 2013-2014, there is a general trend of decreasing prevalence. Therefore, the real decrease is probably greater than that observed. Moreover, given that the risk of HBV perinatal transmission positivity correlates with the viral load of the mother [22], it can be assumed that all women at high risk of HBV transmission are detected, even using a rapid test of low sensitivity.

## 5. Conclusion

The prevalence of HBsAg among women attending antenatal care in Vientiane capital remains high, although it is below the 8% threshold of hyperendemicity. Despite a weak decreasing trend, such prevalence level of chronic HBV infection in pregnant women highlights a persistent risk of HBV perinatal transmission and encourages the strengthening of the birth dose vaccine coverage against hepatitis B in Lao PDR. Getting a clearer understanding of HBV epidemiology in the general Lao population would require other studies like this one from various subsamples of the general Lao population.

## Figures and Tables

**Table 1:** Estimates and significance of the logistic regression model explaining the positivity to HBsAg as a function of age and year. Polynomial terms to the third order were not significant and thus not included in the final model. Probabilities presented here account for potential confounding; see Materials and Methods for further detail.

	Estimate	Std. error	Chisq value	Pr(>Chisq)
Intercept	85.550251	38.790690	—	—
Year	−0.043927	0.019298	5.1992	0.0226
Age	−0.002616	0.007763	0.1138	0.7358
